# Statins reduce all-cause mortality in chronic obstructive pulmonary disease: a systematic review and meta-analysis of observational studies

**DOI:** 10.1186/1465-9921-15-80

**Published:** 2014-07-16

**Authors:** Nobuyuki Horita, Naoki Miyazawa, Ryota Kojima, Miyo Inoue, Yoshiaki Ishigatsubo, Atsuhisa Ueda, Takeshi Kaneko

**Affiliations:** 1 Department of Internal Medicine and Clinical Immunology, Yokohama City University Graduate School of Medicine, 3-9 Fukuura, Kanazawa-ku, Yokohama 236-0004, Japan; 2 Department of Respiratory Medicine, Saiseikai Yokohamashi Nanbu Hospital, Yokohama, Japan; 3 Respiratory Disease Center, Yokohama City University Medical Center, Yokohama, Japan

**Keywords:** Prognosis, Survival, Inflammation, Emphysema

## Abstract

**Background:**

Recent observational studies have suggested that use of statins reduces mortality in patients suffering from chronic obstructive pulmonary disease. However, no meta-analysis has reported the pooled hazard ratio of statins to all-cause mortality.

**Methods:**

We searched for eligible articles using five databases. We included randomized controlled trials and cohort studies written in English using original data reporting the hazard ratio of statins to all-cause, cardiovascular-related, cancer-related, or respiratory-related mortality. A fixed model with the confidence interval method was used. Publication bias was evaluated by funnel plot and Begg’s test, and was corrected using Duval’s trim and fill method. Sensitivity analyses were also conducted.

**Results:**

We included 10 out of 128 articles. The pooled hazard ratio of statins to all-cause mortality involving 16269 patients was 0.81 (95% CI: 0.75-0.86, P < 0.001) with moderate heterogeneity (I2 = 52%, P = 0.032). The sensitivity analysis and funnel plot suggested the existence of publication bias. After three possibly unpublished cohorts were imputed, the pooled hazard ratio of 0.83 (95% CI: 0.78-0.88, P < 0.001) still suggested a favorable prognosis in statin-treated patients. The pooled hazard ratio of statins to cardiovascular-related, cancer-related, and respiratory-related mortality were 0.52 (95% CI: 0.27-1.01, P = 0.052), 0.57 (95% CI: 0.32-1.01, P = 0.056), and 0.55 (95% CI: 0.43-0.78, P < 0.001), respectively, although these results were not conclusive as we could not find a sufficient number of original studies dealing with those forms of mortality.

**Conclusions:**

The use of statins for patients suffering from chronic obstructive pulmonary disease may reduce all-cause mortality. This conclusion should be re-evaluated by a registered large-scale randomized controlled trial.

## Introduction

Chronic obstructive pulmonary disease (COPD) is a pulmonary disease characterized by chronic airflow limitation, often accompanied by systemic inflammation and multiple organ co-morbidities [1,2]. Key medications for treating stable COPD are long- and short-acting bronchodilators. In addition, chronic use of inhaled corticosteroids for patients with advanced COPD and short-course systemic corticosteroids during infective exacerbations of COPD are also commonly prescribed for controlling bronchial inflammation [1]. In the last decade, statins, which are known to inhibit endogenous cholesterol synthesis in hepatocytes by blocking the synthesis of cholesterol [3], have repeatedly been reported to have anti-inflammatory actions and to reduce inflammatory markers such as C-reactive protein, interleukin-6, interleukin-8, and tumor necrotizing factor alpha in COPD patients [2]. A number of recent observational studies have also suggested that statins reduce exacerbation, lung cancer, lung function decline over time, cardiovascular events, and even the mortality of COPD patients [3-19]. A commonly proposed hypothesis is that the anti-inflammatory effect of statins prevents COPD exacerbation, cancer, and lung function decline, which together contribute to better prognosis.

COPD is now the fourth leading cause of death in developed countries [1]. If existing agents, such as statins, actually prevent death from COPD, millions of patients will benefit, because even the current first choice medications modestly reduce the mortality of COPD [20,21]. Three systematic reviews were conducted in 2009 to evaluate the effect of statins on the morbidity and mortality of COPD patients [6,7,22]. However, these systematic reviews did not report on the pooled value for mortality, because only a limited number of original articles existed in 2009, and because these original studies reported outcomes using a variety of measurements such as hazard ratio (HR), odds ratio, and relative risk. Furthermore, no previously published systematic review has sufficiently evaluated the publication bias. Additional studies on this topic have been published in the last five years, and an updated systematic review and meta-analysis has been anticipated. Therefore, the aim of the current systematic review and meta-analysis is to estimate the precise impact of statins on mortality in COPD patients.

## Methods

### Study search and evaluation

Institutional review board approval and patient consent were not required due to the review nature of this study.

Two investigators independently searched for eligible articles using the MEDLINE, EMBASE, BIOSIS, Web of Science, and Cochrane Databases as of October 2013. The following search formula was used for MEDLINE: (“COPD” OR “chronic obstructive airway disease” OR “emphysema” OR “chronic bronchitis” OR “chronic airflow obstruction”) AND (“mortality” OR “prognosis” OR “death” OR “mortalities” OR “prognoses” OR “deaths” OR “survival” OR “survivals”) AND ((“statin” or “statins” OR “fluvastatin” OR “simvastatin” OR “atorvastatin” OR “rosuvastatin” OR “lovastatin” OR “pravastatin” OR “hydoxymethylglutaryl-coA reductase inhibitor”) OR ((“antiplatelet” OR “diuretic” OR “angiotensin converting enzyme inhibitor” OR “ACE inhibitor” OR “angiotensin receptor blocker” OR “beta blocker” OR “antiplatelets” OR “diuretics” OR “angiotensin converting enzyme inhibitors” OR “ACE inhibitors” OR “angiotensin receptor blockers” OR “beta blockers”) AND (“hazard ratio” OR “HR” OR “hazard ratios”))). We used names of cardiovascular medications for the search formula, because a few articles in the authors’ reference list, which mainly reported issues related to cardiovascular medications, described the HR of statins to mortality [13,14]. We used similar words for other databases. Articles in the authors’ reference files were also regarded as candidates.

The eligibility criteria for the current meta-analysis were studies written in English using original data reporting the adjusted HR of statins to all-cause, cardiovascular-related, cancer-related, or respiratory-related mortality. Randomized controlled trials (RCT), prospective and retrospective cohort studies were allowed. Duplicate use of the same data was carefully evaluated. The quality of eligible studies was evaluated using a scale comprising four sub-scales with a maximum of two points for each. The sub-scales were cohort entry, exposure definition, outcome, and cofounding assessment. The scores ranged from 0 to 8, wherein a higher score meant better quality [6].

### Statistics

We used a fixed model with the confidence interval method [23] to estimate pooled HR. HR from RCT and adjusted HR from observational studies were adopted. The heterogeneity of original studies was evaluated with (i) the chi-square distribution test with a rejection region of P = 0.1, and (ii) I^2^ test whereby I^2^ < 0% indicates no heterogeneity, 0% < I^2^ < 25% indicates the least heterogeneity, 25% ≤ I^2^ < 50% indicates mild heterogeneity, 50% ≤ I^2^ < 75% indicates moderate heterogeneity, and 75% ≤ I^2^ indicates strong heterogeneity [24]. A funnel plot and Begg’s rank correlation test using the Kendall test with a rejection region of P = 0.1 were used to evaluate the existence of publication bias [25]. If publication bias was suspected, for a sensitivity analysis, Duval’s trim and fill method was used to estimate the HR that was not affected by the publication bias [26,27]. HR by a fixed model and a random model were compared for sensitivity analysis. Sensitivity analyses were also performed by comparing a variety of subgroups using the rejection region for sensitivity of interaction of P = 0.1 [28]. All analyses were performed in Excel Toukei version 5.0 (SSRI, Tokyo Japan).

## Results and discussion

### Study search

**Figure 1:**
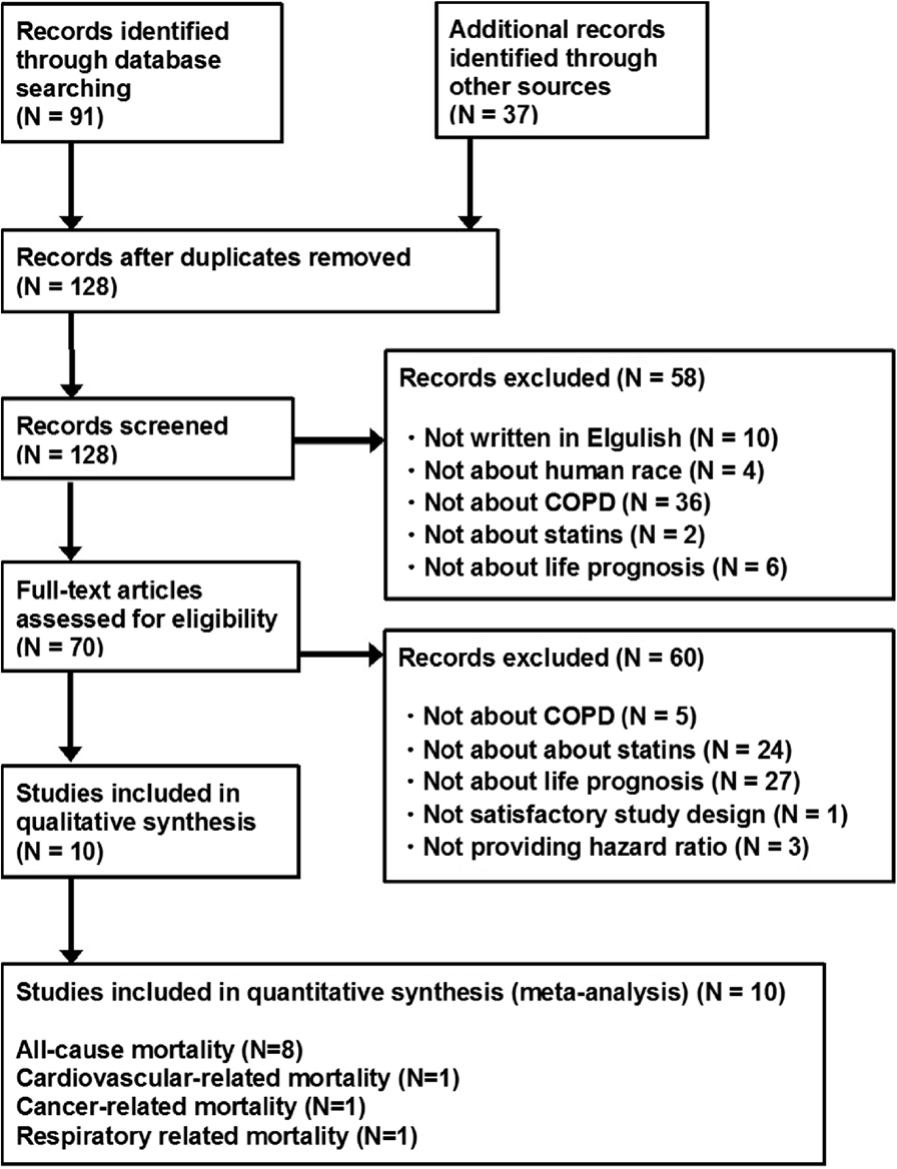
**PRISMA Flow Diagram for study search.** N: number of articles.

Of 128 articles that met the preliminary criteria, we found 10 eligible articles, which included four prospective cohort studies, six retrospective cohort studies, and no RCT (Figure 1, Table 1) [10-19]. Eight articles reported on the HR for all-cause mortality representing nine cohorts, one reported on the HR for cardiovascular-related mortality representing two cohorts, one reported on the HR for cancer-related mortality representing one cohort, and one reported on the HR for respiratory-related mortality representing one cohort. Gestel reported two studies using the same cohort, one for all-cause mortality [11] and the other for cancer-related mortality [12]. Sheng reported two independent cohorts in an article, in which statins were prescribed for primary or secondary prevention [18]. Both all-cause and cardiovascular-related mortality were evaluated in each of the two cohorts [18]. All articles were full length except for Young’s report, which was published in the form of an abstract that evaluated the impact of statins for cause-specific mortality using data from the previously published study by Lawes [16,17]. No study restricted statins to a certain subclass (Table 1).

**Table 1:** Summary of included studies

**Author, Year**	**Design**	**Quality**	**Observed patients**		**Mortality**	**HR (95% CI)**
			**recruitment**	**no**		
Søyseth ’07 [10]	Retro	7	Post exacerbation	854	All	0.57 (0.38-0.87)
Gestel ’08 [11]	Pro	8	Post arterial surgery	1310	All	0.67 (0.52-0.86)
Gestel ’09 [12]					Cancer	0.57 (0.32-1.01)
Rutten ’10 [13]	Retro	6	Population based	2230	All	0.83 (0.65-1.08)
Short ’11 [14]	Retro	6	Post COPD admission	5977	All	0.89 (0.81-0.97)
Bartziokas ’11 [15]	Pro	8	Post exacerbation	245	All	0.85 (0.27-2.69)
Lawes ’12 [16]	Retro	7	Post COPD admission	1687	All	0.69 (0.58-0.84)
Young ’13 [16,17]	Retro	7	Post COPD admission	1687	Resp	0.55 (0.43-0.78)
Sheng ’12 [18]	Retro	7	Population based Primary prevention	1274	All	0.61 (0.43-0.85)
					CV	0.90 (0.35-2.34)
Sheng ’12 [18]	Retro	7	Population based Secondary prevention	443	All	0.58 (0.35-0.97)
					CV	0.32 (0.13-0.77)
Ekström ’13 [19]	Pro	6	Population based On long-term oxygen therapy	2249	All	0.86 (0.72-1.03)

Pro: prospective cohort study. Retro: retrospective cohort study. Quality: higher score indicates better quality and 8 is the maximum score.

All: all-cause mortality. Cancer: cancer-related mortality. CV: cardiovascular-related mortality. Resp: respiratory-related mortality.

Gestel reported two studies using a same cohort. Lawes and Young reported a same cohort.

Ten articles were published during the years 2007-2013. The number of patients in each cohort ranged from 245 to 5977. Without double counting articles by Gestel, Lawes, Young and Sheng [11,12,16-18], 16269 patients were included in our analysis. The quality score of ten articles was in the range six to eight out of eight, which meant that the quality of these observational studies was generally good (Table 1). Two out of two points concerning entry criteria and outcome definition were given for all articles. However, most articles had deficits concerning exposure definition, such as dosage and subclass; and/or concerning confounding assessment, such as sensitivity analysis for statin-related mortality. Three articles with the poorest quality score of six points focused on beta-blockers or other cardiovascular medication, and reported on the HR of statins secondarily. Thus, these three did not report detailed information about statins, though they were originally considered highly-qualified articles [13,14,19].

### All-cause mortality

**Figure 2:**
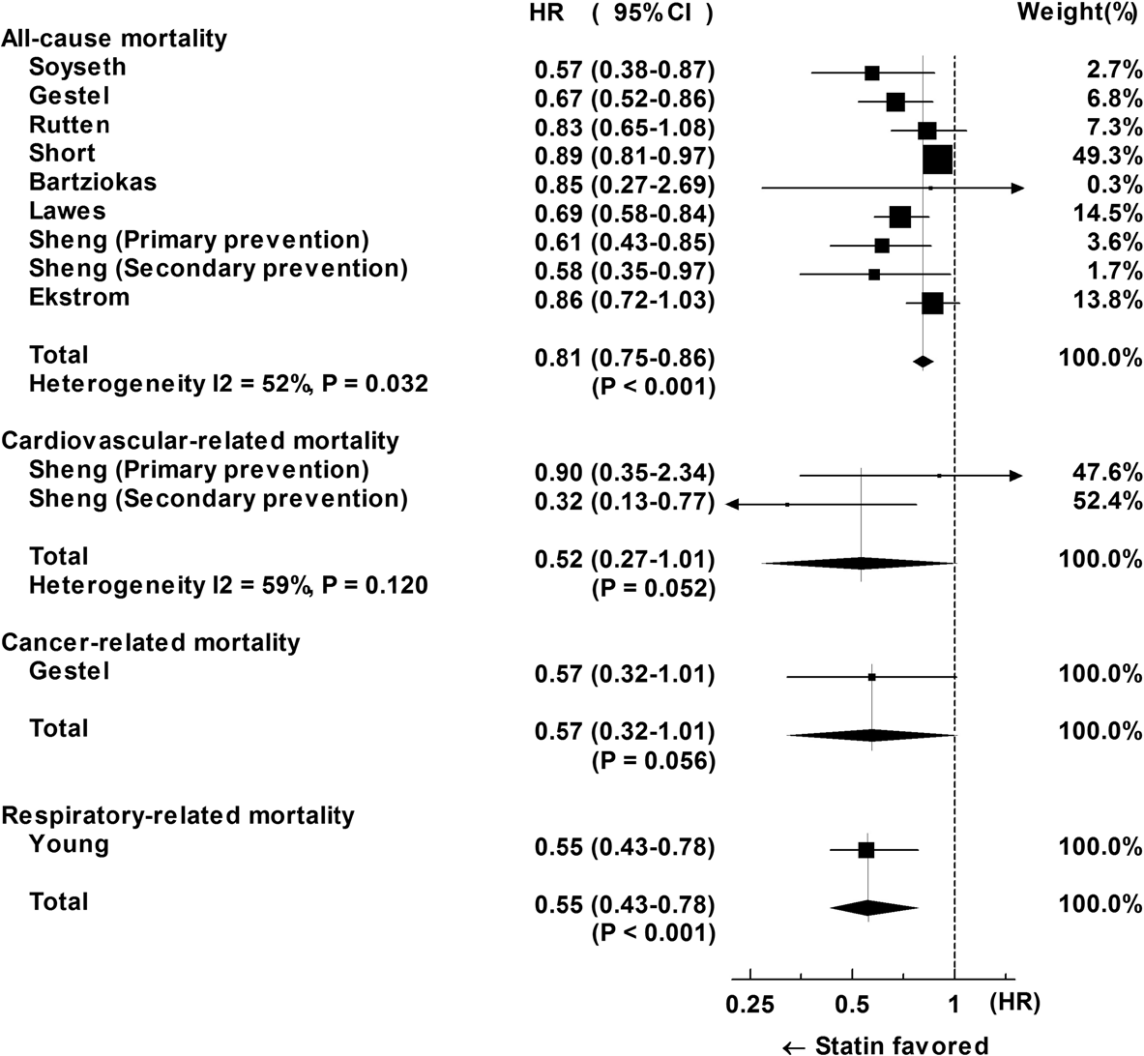
Forest plots for hazard ratio (HR) of statins for mortality.

**Figure 3:**
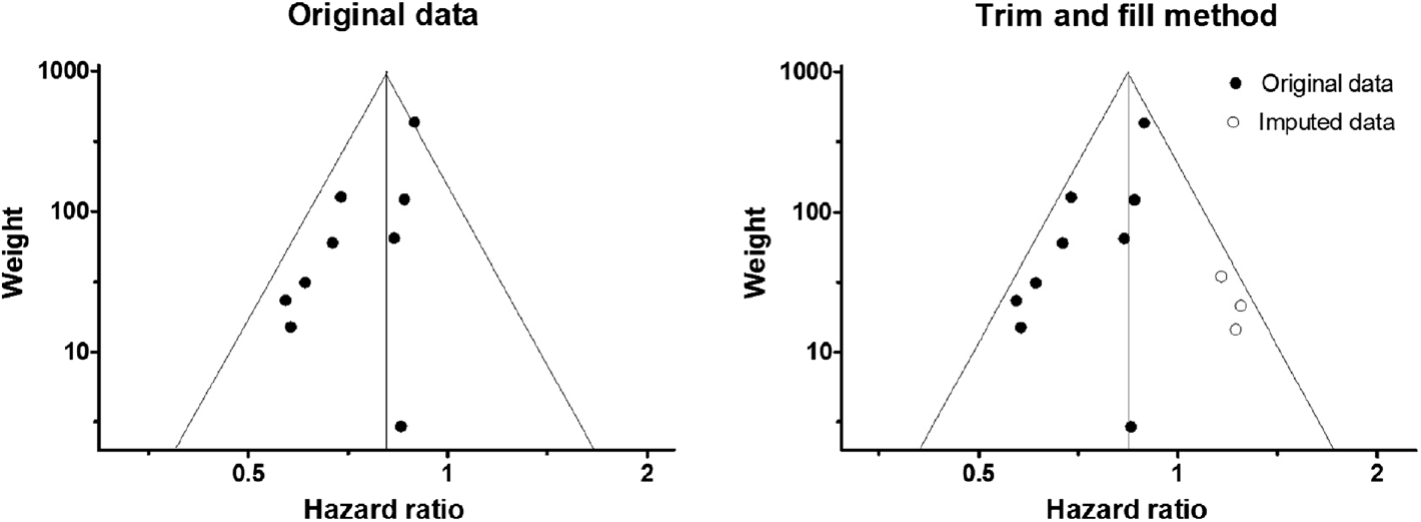
**Funnel plots for studies evaluating all-cause mortality.** Imputed data (open circles) are imaginary data to compensate for a non-symmetric funnel plot. Three open circles and three filled circles were allocated symmetrically with respect to the vertical line.

All of the nine HRs for all-cause mortality by statins were reported as < 1. A fixed-model analysis for nine cohorts evaluating HR of statins to all-cause mortality involving 16269 patients yielded a pooled HR of 0.81 (95% CI: 0.75-0.86, P < 0.001) with significant moderate heterogeneity (I^2^ = 52%; P = 0.032 (<0.1)) (Figure 2). Non-symmetrically displayed cohorts in a funnel plot (Figure 3) could not preclude the existence of publication bias (Figure 3), though a Begg-Kendall rank correlation test (τ = 0.22, P = 0.404 (>0.1)) did not detect it.

### Sensitivity analysis for all-cause mortality

**Figure 4:**
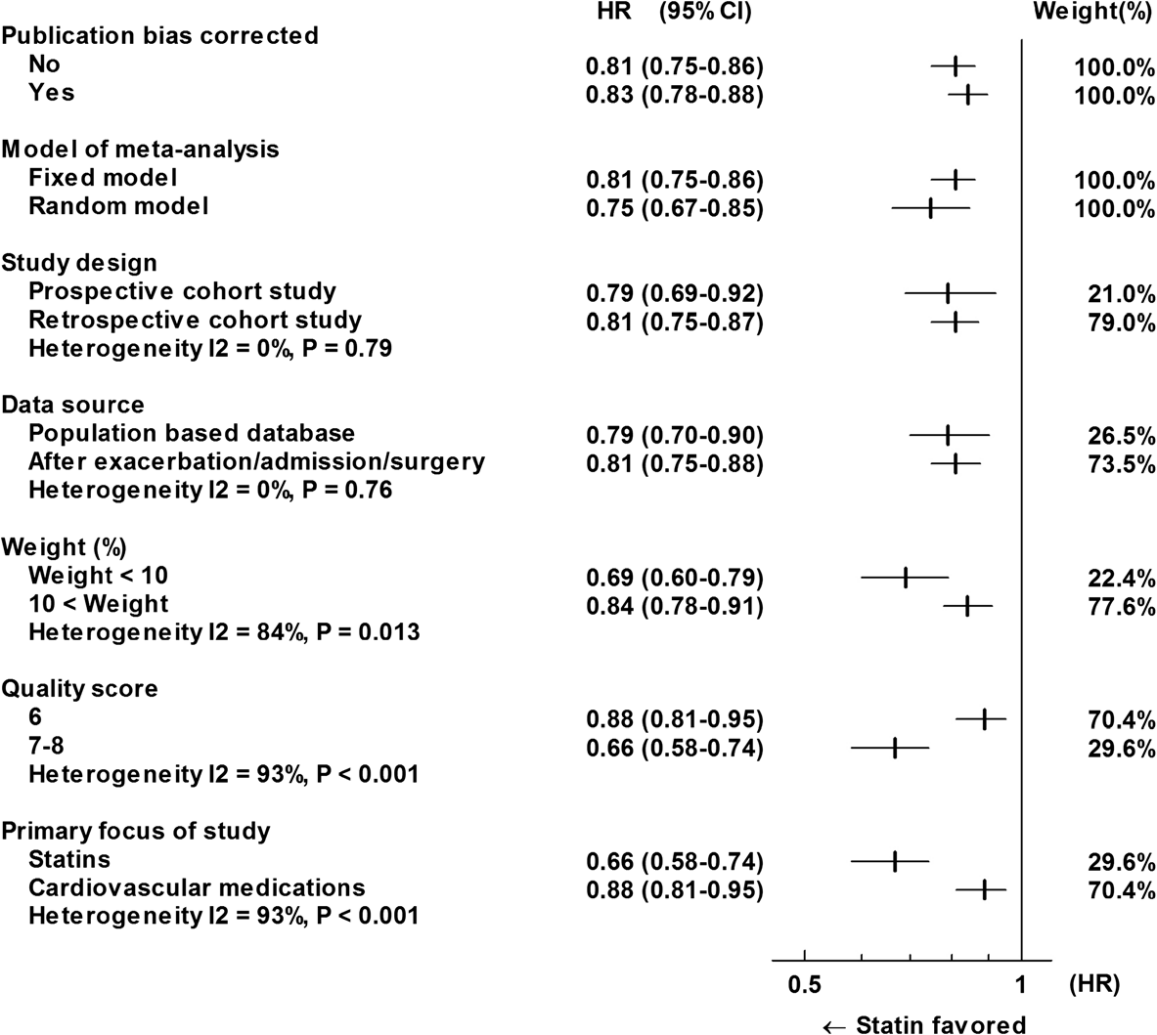
Sensitivity analysis for all-cause mortality.

There were three unreported cohorts according to Duval’s trim and full method. After three possibly unpublished studies were imputed, the pooled HR was slightly shifted toward the null, HR = 0.83 (95% CI: 0.78-0.88, P < 0.001), but it still indicated significantly favorable survival with use of statins (Figures 3 and 4).A random model for all 16269 patients yielded a pooled HR of 0.75 (95% CI: 0.67-0.85, P < 0.001). The result was compatible with that from the fixed model, judged from two 95% CIs for two models overlapping with each other (Figure 4).

A sensitivity analyses with a variety of subgroups was conducted (Figure 4). While the study design and data source did not show significant heterogeneity (P > 0.1), study weight (%) (I^2^ = 84%, P < 0.013 (<0.1)) and quality score (I^2^ = 93%, P = 0.001 (<0.1)) were associated with strong heterogeneity.

### Cardiovascular-related mortality

The HRs for cardiovascular-related mortality were reported for two different cohorts in one article. A fixed-model analysis for two cohorts evaluating the HR of statins to cardiovascular-related mortality involving 1717 patients yielded a pooled HR of 0.52 (95% CI: 0.27-1.01, P = 0.052) (Figure 2) with a marginal significance.

We did not perform a funnel plot analysis, a Begg-Kendall rank correlation test, or a sensitivity analysis, because we could find only two studies.

### Cancer-related mortality

The HR for cancer-related mortality was reported in one article on 1310 patients. The article reported a HR of 0.57 (95% CI: 0.32-1.01, P = 0.056).

We did not perform a meta-analysis, because we could find only one cohort.

### Respiratory-related mortality

The HR for respiratory-related mortality was reported in one article on 1687 patients. The article reported a HR of 0.55 (95% CI: 0.43-0.78, P < 0.001).

We did not perform a meta-analysis, because we could find only one cohort.

## Discussion

In the current meta-analysis, the HR of statins for all-cause mortality in nine cohorts presented in eight articles was 0.81 (Figure 2). The meta-analysis showed moderate heterogeneity, probably due to the publication bias. The HR was slightly increased to 0.83 after three possibly unpublished cohorts were imputed (Figures 3 and 4). The HR of 0.83 seems a much more reserved value compared to most of the original studies. However, the HR for all-cause mortality of 0.83 is still compatible with or even better than the treatment effect by bronchodilators for COPD patients [20,21] and the treatment effect by statins for high-risk primary prevention cohorts [29].

During the study search for the current meta-analysis, we found some articles reporting the effect of statins on all-cause mortality, which we could not include in the analysis due to lack of data on HR. In 2006, Mancini conducted a time-matched nested case-control study of two population-based retrospective cohorts and reported that fully adjusted risk ratios of statins for death ranged from 0.49 to 0.53, depending on the cohort definition [30]. The following year, Ishida researched the correlation between COPD mortality and statin use as expressed by statin sales per capita in an elderly population. Among 47 prefectures in Japan, the correlation coefficient was -0.574 (P < 0.001) [5]. According to Mortensen’s retrospective cohort study with 11212 patients in 2009, current statin use was associated with decreased 90-day mortality with an adjusted odds ratio of 0.51 [31]. In 2012, Lahousse reported that long-term (>2 year) statin use was associated with a 39% reduction of all-cause mortality after adjusting for confounding variables. Despite the demonstrable results, the report was not included in the current meta-analysis as it does not report in the form of HR, but of odds ratio [32]. Although we should adopt a careful attitude toward these results due to the limitations of the study design, it is noteworthy that all of the studies above and the original researches included in the current meta-analysis consistently reported a favorable life prognosis by the use of statins. In addition, favorable effects for systemic inflammation, lung mechanics, quality of life, exacerbation, and admission were also confirmed [2-4].

The role of systematic inflammation in COPD has recently been emphasized [1,2]. The currently used medications including inhaled corticosteroids are known to reduce symptoms and airflow obstruction. However these medications have limited effect on the natural history of COPD [1]. On the other hand, the anti-inflammatory effects of statins on pulmonary and systemic inflammation have been repeatedly reported [2,3]. In the current analysis, it is reasonable to assume that hypercholesterolemia was more prevalent among statin users compared to non-statin users and that statin users were at high risk of a cardiovascular event and death. However, the existence of hypercholesterolemia and values of cholesterol and/or triglyceride were not always adjusted in the original studies [10-19]. In short, even though statin users may have hypercholesterolemia, the current meta-analysis indicated improved life prognosis by the use of statins. This effect could not be fully explained by cardiovascular event prevention. The anti-inflammatory effect of statins may prevent death along with cardiovascular event prevention. The anti-inflammatory effect of statins was also reported to be relevant to hospitalization, exacerbation, intubation, and decline of lung function in COPD cases [2-4,7,22]. This may explain why statins reduce mortality.

A funnel plot and sensitivity analysis suggested the existence of publication bias (Figures 3 and 4). Publication bias is caused by the tendency of researchers and editors to publish the reporting of positive results. While relatively small weighted studies showing favorable results are likely to be published, those showing inconclusive or harmful results are not likely to be published. To avoid selective reporting of trials, RCTs with clinical trial registration is a reasonable solution [33]. Results from RCTs such as the ongoing “STATins in COPD Exacerbations” are anticipated [34]. Studies with high quality score and studies focusing on statins have indicated significantly lower HR (Figure 4), though interpretation is difficult. A simple interpretation is that the HR of 0.66 reported by studies with quality scores of 7 or 8 and by studies focusing on statins is more reliable than the HR of 0.88 reported by the other studies with a quality score of 6. Another interpretation is that there was a publication bias or a selective outcome reporting bias. In other words, authors who observed favorable HR by statins tended to make reports concentrating on statins. As mentioned in the results section above, three articles with near-null HR focused on cardiovascular medications and did not provide a detailed description about statins, which resulted in poor quality scores despite the careful study design [13,14,19]. In our opinion, it is difficult to conclude that results from three studies of lower quality (score = 6) not focusing on statins are less reliable than those from the other studies of higher quality (score = 7 or 8) focusing on statins. The important thing is that pooled HR suggested a statin-favorable result even with the low quality studies that suggested near-null HR.

Besides publication bias, our study has some limitations. First, none of the included studies were RCTs, but observational studies. Although meta-analysis with an RCT is usually preferred, meta-analysis with non-RCT studies is commonly accepted and the number of published meta-analyses with observational studies has increased [35]. That is because an RCT is not always feasible and timely, and because observational studies often yield effects estimates comparable to RCT [35,36]. Furthermore, patients satisfying strict inclusion criteria for RCT do not always reflect real world patients with multiple co-morbidities. We believe that the results from the current meta-analysis are trustworthy, because we conducted the current study following guideline for meta-analysis of observational studies [36]. Second, HR of 0.52, 0.57, and 0.55 for cardiovascular-related, cancer-related, and respiratory-related mortality were not conclusive due to the limited number of available studies. The small number of the included original studies is also a limitation for evaluating all-cause mortality. Third, some may think that the heterogeneity among studies may detract from the reliability of the current meta-analysis. However, we believe that the observed moderate heterogeneity of I^2^ = 52% is acceptable for a meta-analysis. Furthermore, the consistency between the results from HRs by fixed-model (HR = 0.81, 95% CI 0.75-0.86) and random-model (HR = 0.75, 95% CI 0.67-0.85) makes the results reliable despite the heterogeneity.

## Conclusion

In conclusion, even after possibly unpublished studies were imputed, the pooled HR of statins to all-cause mortality was 0.83 (95% CI: 0.78-0.89, P < 0.001). The HR of 0.83 is a discreet value compared to many previous observational studies, but is still encouraging. HR for cardiovascular-related, cancer-related, and respiratory-related mortality was not sufficiently evaluated in the current analysis as we could not find a sufficient number of original studies dealing with those forms of mortality. Although this meta-analysis and previous original studies have common limitations in their observational nature, these studies have presented meaningful results. The possibly very propitious treatment effect of statins, which may lead to a paradigm shift in the treatment of COPD, should be re-evaluated by a large-scale RCT.

## Abbreviations

COPD: Chronic obstructive pulmonary disease; HR: Hazard ratio; RCT: Randomized controlled trial; 95% CI: 95% confidence interval.

## Competing interests

None of the investigators declare any real or perceived conflicts of interest pertaining to the subject of this manuscript.

## Authors’ contributions

All authors contributed to the conception, design, data acquisition, analysis, interpretation, drafting, revising, and final approval of the manuscript. NH who is a statistician served as a principal investigator (guarantor). NM, and RK provided interpretation of data and drafting. MI worked for study search. YI, AU, and TK provided study management.

## Financial statement

No support in the form of grants, gifts, equipment, and/or drugs was provided.
